# Titanium dioxide particle – induced goblet cell hyperplasia : association with mast cells and IL-13

**DOI:** 10.1186/1465-9921-6-34

**Published:** 2005-04-13

**Authors:** Mi-Hyun Ahn, Chun-Mi Kang, Choon-Sik Park, Sang-Jun Park, Taiyoun Rhim, Pyeong-Oh Yoon, Hun Soo Chang, Soo-Ho Kim, Hiroko Kyono, Kwang Chul Kim

**Affiliations:** 1 Genome Research Center for Allergy and Respiratory disease, Soonchunhyang University Hospital, Bucheon, Korea; 2 National Institute of Industrial Health, Kawasaki, Japan; 3 Department of Pharmaceutical Sciences, University of Maryland School of Pharmacy, Baltimore, Maryland, USA

**Keywords:** goblet cells, Muc5ac, particle, IL-13, mast cell, dexamethasone, cyclophosphamide

## Abstract

**Background:**

Inhalation of particles aggravates respiratory symptoms including mucus hypersecretion in patients with chronic airway disease and induces goblet cell hyperplasia (GCH) in experimental animal models. However, the underlying mechanisms remain poorly understood.

**Methods:**

To understand this, the numbers of goblet cells, Muc5ac (+) expressing epithelial cells and IL-13 expressing mast cells were measured in the trachea of sham or TiO_2 _particles – treated rats using periodic acid-Schiff, toluidine blue and immunohistochemical staining. RT-PCR for Muc-1, 2 and 5ac gene transcripts was done using RNA extracted from the trachea. Differential cell count and IL-13 levels were measured in bronchoalveolar lavage (BAL) fluid. In pretreatment groups, cyclophosphamide (CPA) or dexamethasone (DEX) was given before instillation of TiO_2_. TiO_2 _treatment markedly increased Muc5ac mRNA expression, and Muc5ac (+) or PAS (+) epithelial cells 48 h following treatment.

**Results:**

The concentration of IL-13 in BAL fluids was higher in TiO_2 _treated – rats when compared to those in sham rats (p < 0.05). Pretreatment with cyclophosphamide (CPA) decreased the number of neutrophils and eosinophils in BAL fluid of TiO_2 _treated – rats (p < 0.05), but affected neither the percentage of PAS (+) cells, nor IL-13 levels in the BAL fluids (p > 0.05). In contrast, pretreatment with dexamethasone (DEX) diminished the percentage of PAS (+) cells and the levels of IL-13 (p < 0.05). TiO_2 _treatment increased the IL-13 (+) mast cells (p < 0.05) in the trachea, which was suppressed by DEX (p < 0.05), but not by CPA pretreatment (p > 0.05). In addition there were significant correlations of IL-13 (+) rate of mast cells in the trachea with IL-13 concentration in BAL fluid (p < 0.01) and with the percentage of Muc5ac (+) cells in the sham and TiO_2 _treated rats (p < 0.05).

**Conclusion:**

In conclusion, TiO_2 _instillation induces GCH and Muc5ac expression, and this process may be associated with increased production of IL-13 by mast cells.

## Background

Excessive mucus secretion is one of the major clinical manifestations of chronic airway diseases such as asthma, chronic bronchitis, and cystic fibrosis [1]. The excessive mucus is attributed to goblet cell hyperplasia (GCH) and submucosal gland hypertrophy, which are hallmarks of airway remodeling in chronic airway diseases [2,3]. Air pollution aggravates respiratory symptoms in patients with chronic airway diseases. Chronic obstructive pulmonary disease (COPD) patients living in communities exposed to high levels of air pollution have faster rates of decline in lung function than patients living in areas with low pollution [4]. The level of environmental particles is also positively correlated with exacerbation of asthma [5].

Airborne particulate matter less than 10 μm in aerodynamic diameter (PM10) is a complex mixture of organic and inorganic compounds containing sulfates and various metals such as aluminum, calcium, copper, iron, lead, magnesium, titanium, and zinc [6]. Clinically, PM10 particles are thought to provoke airway inflammation with the release of mediators that are capable of exacerbating lung disease in susceptible individuals [5,7]. This assumption is based on experimental evidence of airway inflammation following direct instillation or inhalation of PM10 particles in animal models [8]. Furthermore, inhaled particles directly stimulate macrophages and epithelial cells to produce inflammatory cytokines such as TNF-α, GM-CSF and IL-8 [9,10], which induce neutrophil- and eosinophil-mediated airway inflammation, and eventually lead to GCH. Recently, particle exposure favors the antigen – sensitized lung toward Th2 environment with over secretion of IL-13, IL-4 [11] and IL-5 [12]. Beside the inflammatory cell mediated – GCH, IL-13 directly induces GCH and Muc5AC gene expression through the signaling of IL-4Rα and IL-13Rα [13,14]. Therefore, we hypothesized that particles induce GCH via over-production of IL-13 by recruited inflammatory cells.

Titanium dioxide (TiO_2_) particles, one component of PM10, are found in dusty workplaces such as industries involved in the crushing and grinding of the mineral ore rutile [15]. It was reported that 50% of TiO_2_-exposed workers had respiratory symptoms accompanied by reduction in pulmonary function [16]. Because acute and chronic exposure to TiO_2 _particles induces inflammatory responses in the airways and alveolar spaces of rats [17,18], TiO_2 _– instilled rat may be a good model to study the particle induced – airway injury. In this study, we evaluated the role of neutrophilic and eosinophilic inflammation by pretreatment with cyclophosphamide inducing neutropenia [19] and the association of IL-13 by pretreatment with dexamethasone suppressing IL-13 gene expression [20].

## Methods

### Treatment protocols

Particles of TiO_2 _(mean diameter = 0.29 μm, DuPont, Wilmington, DE) were suspended in endotoxin-free saline. The endotoxin concentration of the TiO_2 _suspension was less than <0.32 EU/ml as measured with a limulus amebocyte lysate kit (QCL-1000; BioWhittaker, Inc., Walkersville, MD). Seven-week-old male Sprague-Dawley rats (Charles River Technology Inc.) received a single intratracheal instillation of homogeneous suspension of TiO_2 _particles (4 mg/kg in 200 μl of endotoxin free water). In a pretreatment group, cyclophosphamide (CPA) (100 mg/kg, i.p.) was given 5 days before instillation of TiO_2 _and a second injection of CPA (50 mg/kg, i.p.) 1 day before TiO_2 _instillation. In the second pretreatment group, dexamethasone (DEX) (0.25 mg/kg, i.p.; Sigma, St. Louis, MO) was administered 24 h before TiO_2 _instillation. The Institutional Animal Care and Use Committee of Soonchunhyang University approved the study protocols.

### Preparation of lung tissues and morphological analysis

Rats were sacrificed at 4, 24, 48 and 72 hr after TiO_2 _instillation by being anesthetized with pentobarbital sodium (65 mg/kg, i.p.) and bronchoalveolar lavage (BAL) was performed by 5 times instillation of 1 ml normal saline and gentle retrieval. Cell numbers were measured using a hemacytometer and differential cell counts were performed on slides prepared by cyto-centrifugation and Diff-Quik staining (Scientific Products, Gibbstowne, NJ). Immediately following BAL, the trachea was snap-frozen for RNA extraction or fixed with 4% paraformaldehyde in PBS and embedded in paraffin. The tissues were subjected to periodic acid-Schiff (PAS) and toluidine blue staining to permit measurement of goblet cells and mast cells, respectively. Morphometric analysis was performed under light microscopy at ×400 magnification. PAS positive epithelial cells and total epithelial cells were counted on the length of 250 μm basement membrane at each of four predetermined sites (12, 3, 6, 9 o'clock; 12 o'clock was the membranous portion) using a soft program (Nikon DXM 1200, Nikon Inc. N.Y. USA & Image Pro Plus 4.01 software, Media Cybernetics, Maryland, USA). Results are expressed as the percentage of goblet cells among the epithelial cells. Mast cells in the airway wall were counted on the membranous portion. The results are expressed as the number of cells staining positive for toluidine blue per area of 0.01 mm^2^.

### Reverse transcription-polymerase chain reaction (RT-PCR)

Total RNA was isolated using the modified guanidium thiocyanate-phenol-chloroform extraction method [21]. DNase I (10,000 U/ml; Stratagene, La Jolla, CA)-treated RNA was reverse-transcribed by incubating with 0.5 mM dNTP, 2.5 mM MgCl_2_, 5 mM DTT, 1 μl of random hexamer (50 ng/μl) and SuperScript II RT (200 unit/μl; Life Technologies, Grand Island, NY) at 42°C for 50 min, and heat inactivated at 70°C for 15 min. cDNA was aliquoted into tubes containing specific primer pairs for rat GAPDH, Muc1, Muc2 and Muc5ac genes for amplification (300, 403, 421, and 382-bp fragments, respectively). Nucleotide sequences of the primers were as follows. GAPDH-forward ; 5'GGCATTGCTCTCAATGACAA3', GAPDH-reverse; 5'AGGGCCTCTCTCTTGCTCTC3', Muc1-forward; 5' AGAGCTATGGGCAGCTGG 3', Muc1-reverse; 5' ACTACCCCAGTGTCCCTC 3', Muc2-forward; 5' TACTGCTGATGACTGTAT 3', Muc2-reverse; 5'GGCCACAGGCCTGATACT3', Muc5ac-forward; 5' TACAAGCCTGGTGAGTTC 3', Muc5ac-reverse; 5' TCACAGTGCAGCGTCACA 3'. Amplification was performed for 40 cycles (one cycle: 1 min at 94°C, 1 min at 52°C, and 1 min at 72°C) with initial denaturation at 94°C for 5 min and a final extension at 72°C for 10 min.

### Immunohistochemical identification of Muc5ac-expressing epithelial cells and IL-13-expressing cells

Muc5ac-positive (+) epithelial cells and IL-13-positive (+) cells were identified by immunohistochemical staining. Three-micron tissue sections of the trachea were treated with 0.3% H_2_O_2_-methanol for 20 min to block endogenous peroxidase, and then incubated at 4°C overnight with anti-rat Muc5ac mouse monoclonal antibody (1:200 dilution; Neomarkers, Fremont, CA) or biotinylated anti-rat IL-13 antibody (1:5 dilution; Biosource, Camarillo, CA). After the slides had been incubated with avidin-biotin peroxidase complex (ABC kit, Vector Laboratories, Burlingame, CA), color was developed with 3,3'-diaminobenzidine tetrachloride (DAB, Zymed Laboratories, South San Francisco, CA). The Muc5ac expressing epithelial cells and total epithelial cells were counted on the length of 250 μm epithelial basement membrane at each of four predetermined sites (12, 3, 6, 9 o'clock; 12 o'clock was the membranous portion). Results are expressed as the percentage of Muc5ac (+) cells among the epithelial cells. IL-13 (+) cells was counted on the membranous portion in the same way as mast cells were counted. The results are expressed as the positive rate of mast cells for IL-13 stain per area of 0.01 m^2^.

### Measurement of IL-13 concentration in BAL fluids

The levels of IL-13 in the BAL fluids were measured with a quantitative sandwich enzyme-linked immunoassay kit (Biosource, Camarillo, CA). The lower limit of detection was approximately 1.5 pg/ml. Values below this limit were considered as zero for statistical analysis. Inter- and intra-assay coefficients of variance were less than 10%.

### Statistical analysis

Differences between independent samples were compared using the Spearman test for continuous data. If differences were found significant, the Mann-Whitney U test was applied to compare differences between two samples. Differences were considered significant when the p value was less than 0.05. Results are expressed as means ± standard error of the mean (SEM) unless otherwise stated. The correlations were analyzed between the ratio of Muc5ac (+) expressing epithelial cell and the concentration of IL-13 in BAL fluid and the number of mast cell and the IL-13 positive rate of mast cells by Spearman's non-parametric correlation using SPSS (version 10.0, Chicago, USA)

## Results and Discussion

### Expression of Muc gene transcripts in the trachea of TiO_2 _or saline – instilled rats

**Figure 1:**
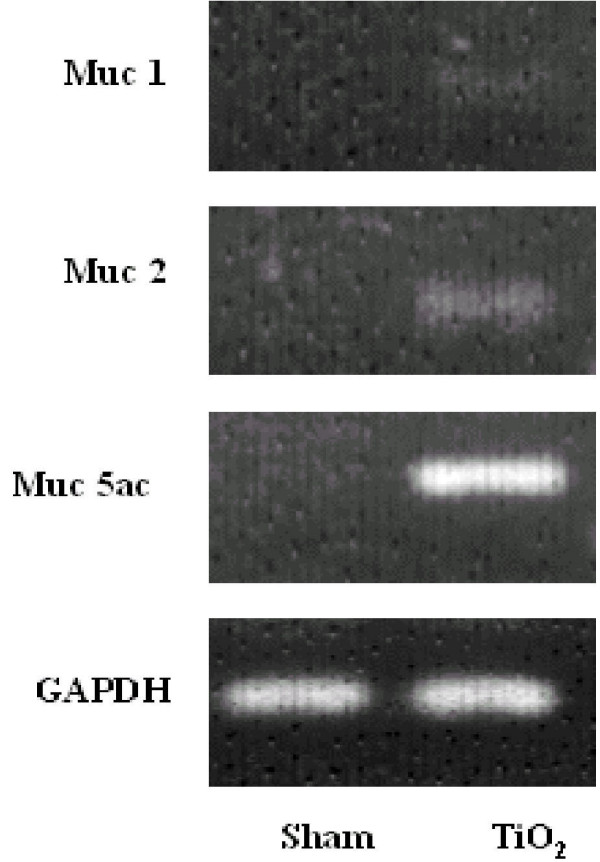
The expression of Muc1, Muc2 and Muc5ac mRNA in TiO_2 _treated rats. Rats were treated with TiO_2_, as described in Methods. Twenty-four hours after treatment, the levels of the Muc gene transcripts in the trachea were quantified using RT-PCR. GAPDH was used to ensure an equal loading of RNA samples. This figure is representative of 4 experiments.

Total RNA was extracted from the trachea 24 h following treatment with saline or TiO_2 _and analyzed for Muc1, Muc2, and Muc5ac transcripts by RT-PCR. As shown in Figure 1, Muc1, Muc2 and Muc5ac mRNAs were practically undetectable in sham-treated rats. In contrast, TiO_2 _treatment markedly increased Muc5ac mRNA, but only modestly increased Muc2 mRNA. Muc1 mRNA was not seen in TiO_2_-treated rats.

### The effect of TiO_2 _instillation on Muc5ac-positive and PAS-positive epithelial cells in trachea

**Figure 2:**
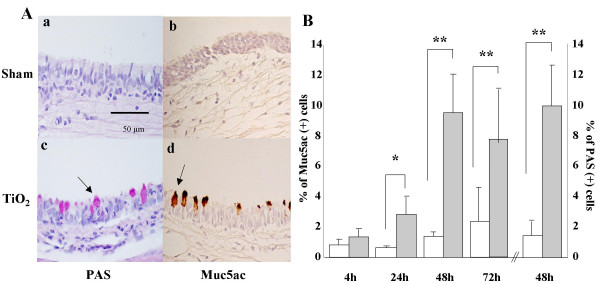
Light microscopic analysis of the trachea and the percentage of Muc5ac, PAS (+) epithelial cells. Rats were treated intratracheally with saline or TiO_2_, and the tracheas were prepared for morphometric analysis of PAS (+) and Muc5ac (+) cells as described in Methods. **A**. Histology of trachea 24 hr after saline or TiO_2 _treatment. PAS (+) cells were stained red whereas Muc5ac (+) cells dark brown. Note that the trachea obtained from the saline-treated group contained little or no PAS (+) (Aa) or Muc5ac (+) cells (Ab) while the trachea from TiO_2_-treated group contains significant number of PAS (+) (Ac) and Muc5ac (+) cells (Ad). **B**. Time (4, 24, 48,72 h) dependent change in the percentage of Muc5ac (+) cells following saline (open bar, n = 8) or TiO_2 _treatment (closed bar, n = 8). Note that the percentage of PAS (+) cells was similar to that of Muc5ac (+) cells at 48 hr after TiO_2 _instillation. * p < 0.05, ** p < 0.01 as compared with the saline treated group.

Rats were given a single intratracheal instillation of saline or TiO_2 _and the percentage of Muc5ac-positive (Muc5ac (+)) and PAS-positive (PAS (+)) epithelial cells were measured. At 24 h after saline instillation, almost no PAS (+) or Muc5ac (+) epithelial cells were found in the trachea (Fig. 2Aa, b). TiO_2 _instillation, however, induced PAS (+) or Muc5ac (+) cells in the trachea at 24 h (Fig. 2Ac, d). The percentage of Muc5ac (+) cells was significantly higher at 24 hr (p < 0.05) and further increased (Fig. 2B) in TiO_2 _– instilled rats and maintained until 72 h when compared with those of sham rats (p < 0.01). The percentage of PAS (+) cells was very similar to that of Muc5ac (+) cells at 48 h after TiO_2 _instillation (Figure 2B).

### Effects of cyclophosphamide and dexamethasone on the number of inflammatory cells and IL-13 levels in BAL fluid of TiO_2_-treated rats

**Figure 3:**
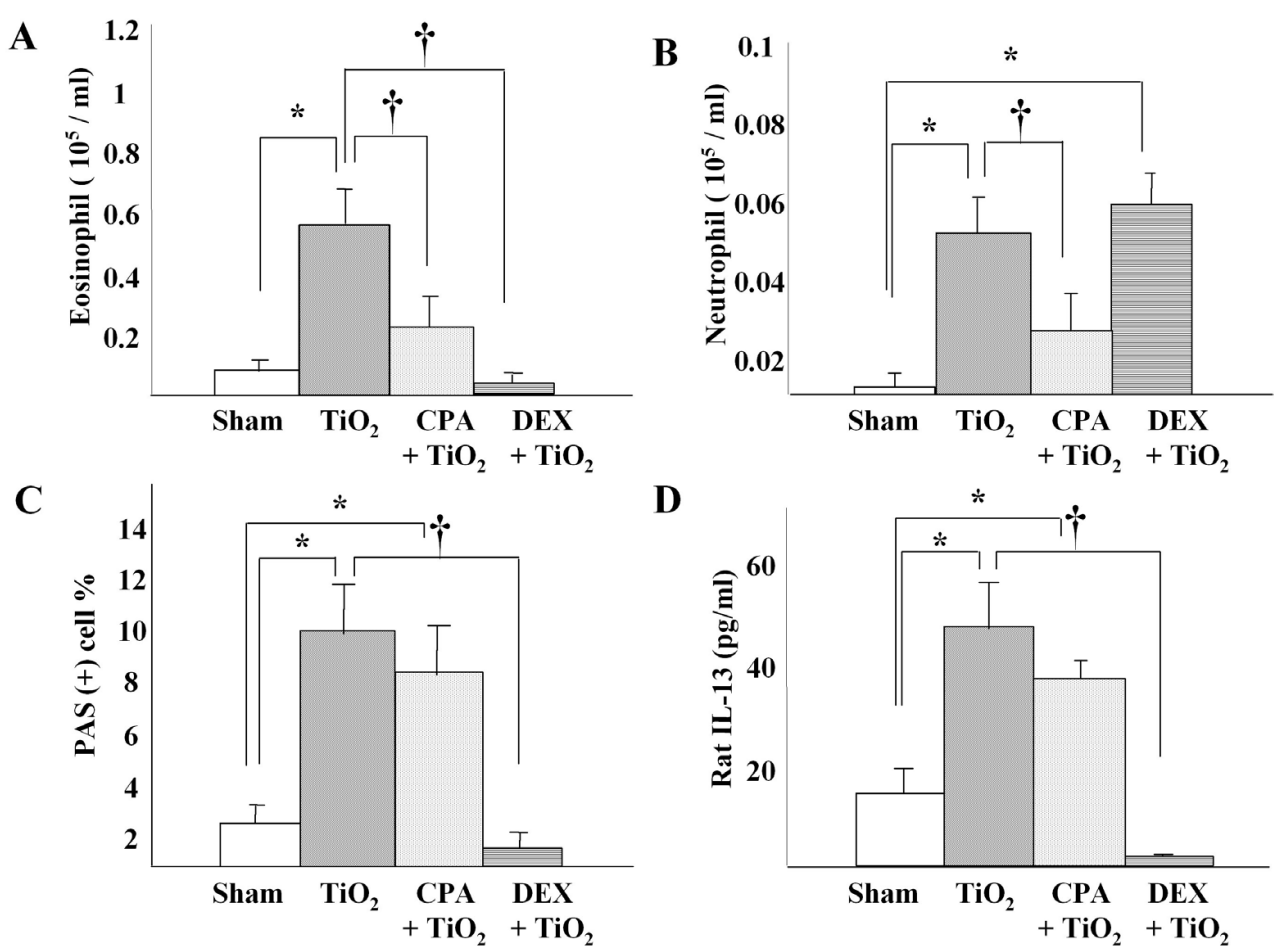
The cell distribution in BAL fluid of TiO_2 _instilled rats with or without pretreatment. Rats were pretreated with CPA (n = 6) or DEX (n = 6) and then treated intratracheally with TiO_2_. Saline (n = 8) or TiO_2 _(n = 8) was treated without pretreatment. At 48 h post-treatment, BAL fluids were collected and analyzed for the numbers of eosinophils (A), neutrophils(B), and the levels of IL-13(D). PAS (+) cells (C) were measured in the trachea as described in Methods. * p < 0.05 as compared with saline – treated group, † p < 0.05 as compared with TiO_2 _– treated group.

**Figure 4:**
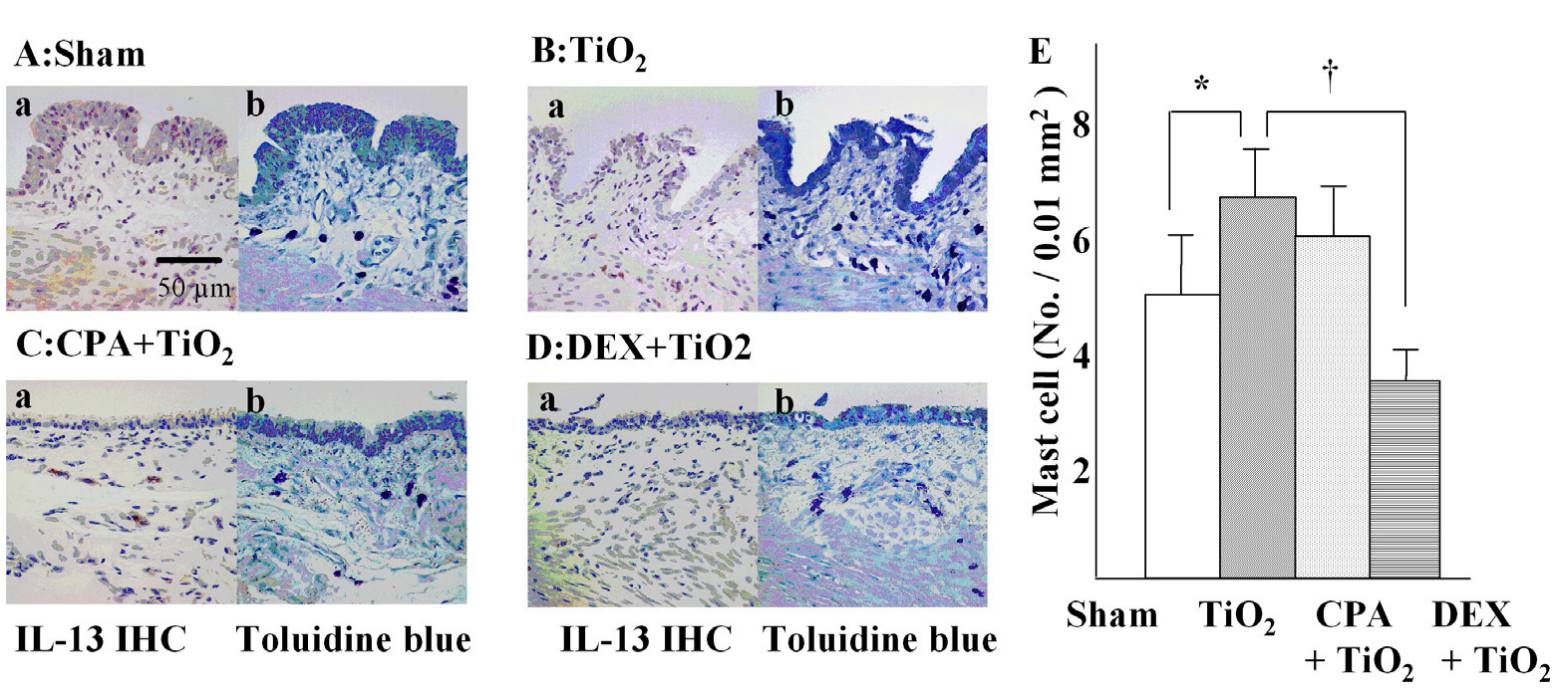
The effects of cyclophosphamide (CPA) or dexamethasone (DEX) on the IL-13 (+) expressing cells. Rats were pretreated intratracheally with saline (Fig. A, E ; n = 8), CPA (Fig. C, E ; n = 6) or DEX (Fig. D, E ; n = 6) prior to treatment with TiO_2 _Eight rats were treated with TiO_2 _alone (Fig. B, E ; n = 8) as described in Methods. At 48 h post-treatment, IL-13 (+) cells are stained brown whereas toluidine blue (+) mast cells are stained dark purple. Note that saline – treated group contained little or no IL-13 (+) cells (Aa) in spite of the presence of mast cells (Ab). TiO_2_-treated group showed significantly increasing numbers of mast cells when compared with sham group (E) and the mast cells (Ba) showed strong positivity for IL-13 protein (Bb). CPA pretreatment did not affect the TiO_2 _induced-increase in the number of IL-13 (+) cells (Ca) or mast cells (Cb & E). On the other hand, DEX pretreatment significantly decreased the number of mast cells (Db & E) and reduced the IL-13 (+) cells (Da). * p < 0.05 as compared with saline treated group, † p < 0.05 as compared with TiO_2 _treated group.

The numbers of eosinophils and neutrophils are markedly increased in the BAL fluids at 48 h after TiO_2 _instillation when compared with those in saline-treated rats (p < 0.05, respectively) (Fig. 3A and 3B). Also, the levels of IL-13 in BAL fluids were significantly higher in TiO_2 _– treated rats than those of sham rats at 48 h after treatment (p < 0.05) (Fig. 3D). Pretreatment with CPA prior to TiO_2 _instillation significantly decreased the numbers of neutrophils and eosinophils in BAL fluids when compared with those in rats at 48 h after treatment with TiO_2 _alone (p < 0.05, Fig. 3A &3B). Pretreatment with CPA, however, did not affect both the ratio of PAS (+) cells in the trachea and the IL-13 levels in BAL fluids of TiO_2_-treated rats (p > 0.05, Fig. 3C &3D). Pretreatment with DEX prior to TiO_2 _instillation significantly decreased the number of eosinophils in BAL fluid (p < 0.05, Fig. 3A), the ratio of PAS (+) cells in the trachea (p < 0.05, Fig 3C) and the levels of IL-13 in BAL fluid (p < 0.05, Fig. 4D) compared with those of rats instilled by TiO_2 _alone.

### Effects of cyclophosphamide and dexamethasone on the number and IL-13 expression of mast cells in TiO_2_-treated rats

Toluidine blue – stained mast cells were observed in and around the muscle layer of the trachea in saline-treated rats. The shape of the cells was relatively round with a single nucleus and a large cytoplasm containing granules (Fig. 4Ab). In TiO_2_-instilled rats, some mast cells showed an elongated and branching shape of the cytoplasm (Fig. 4Bb). The trachea of the saline-treated group contained no IL-13 (+) cells (Fig. 4Aa) in spite of the presence of mast cells (Fig. 4Ab). TiO_2_-instilled rats increased the number of mast cells when compared with the saline control group (p < 0.05, Figs. 4Bb and 4E). Serial section slides of the trachea showed that IL-13 protein was expressed exclusively on the mast cells in TiO_2 _– treated rats (Fig. 4Ba). CPA pretreatment did not affect the TiO_2_-induced increase in the number of toluidine blue (+) mast cells positive for IL-13 (p > 0.05, Fig. 4Ca, 4Cb &4E). However, DEX pretreatment significantly decreased the number of toluidine blue (+) mast cells expressing IL-13 compared to those of TiO_2 _– treated rats (p < 0.05, Fig. 4Da, 4Db &4E).

### The correlation between the number of IL-13 expressing mast cells, the concentration of IL-13 in BAL and Muc 5ac positive epithelial cells in the airway

**Figure 5:**
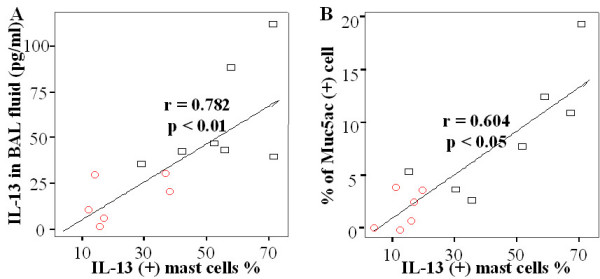
The correlation of the IL-13(+) mast cells with Muc5ac(+) epithelial cells and the IL-13 concentration. The percentage of IL-13 (+) mast cells was correlated with concentration of IL-13 in BAL fluid (r = 0.782, p < 0.01) and the percentage of Muc5ac (+) cells (r = 0.604, p < 0.05) (open circle; sham, open square; TiO_2 _– instilled rats).

The number of mast cells in the trachea was significantly correlated with percentage of Muc5ac (+) epithelial cells and concentration of IL-13 in BAL fluid of TiO_2 _– treated (n = 7) and sham (n = 6) rats (p < 0.001 and p < 0.0001, respectively, Table 1). However, the number of eosinophil and neutrophils in BAL fluids were correlated with neither the percentage of Muc5ac (+) epithelial cells nor the concentration of IL-13 in BAL fluid (p > 0.05, Table 1). In addition, there were significant correlations of IL-13 (+) rate of mast cells in the trachea with IL-13 concentration in BAL fluid (r = 0.782, p < 0.01, Fig. 5A) and with the percentage of Muc5ac (+) cells in the sham and TiO_2 _treated rats (r = 0.604, p < 0.05, Fig 5B).

**Table 1:** The correlation of Muc5ac(+) cells or the IL-13 concentration with the number of inflammatory cells. The correlation between percentage of Muc5ac (+) epithelial cells or concentration of IL-13 in BAL fluid and number of eosinophil, neutrophil and mast cell in sham (n = 6) and TiO_2 _– instilled rats (n = 7).

Correlation (ρ)	Eosinophils No. in BAL fluid	Neutrophils No. in BAL fluid	Mast cells No. in trachea
% of Muc5ac (+)	0.156 (p = 0.549)	-0.195 (p = 0.438)	0.813 (p = 0.001*)
Concentration of IL-13 in BAL fluid	0.447 (p = 0.138)	0.193 (p = 0.57)	0.903 (p = 0.0001**)

* p < 0.05, ** p < 0.01

## Discussion

Although air pollution contains heavy metallic environmental particles that increases morbidity and mortality of the patients with chronic airway diseases [4,5], the underlying mechanisms of mucus hyperproduction causing airway obstruction has not been revealed in detail. In this study, we demonstrated that a single instillation of TiO_2 _is able to induce GCH within 24 h. The TiO_2_-induced GCH is associated with a dramatic increase in Muc5ac gene and protein expression in the present study (Figure 1 &2). Up regulation of Muc5ac gene in TiO_2 _– induced GCH is thought to be a common pathway in the process of GCH because MUC5AC has been demonstrated to be a major MUC gene during the process of GCH observed in the other non-particulates experimental model of airway diseases [22-25] and the asthmatics [26]. GCH is known as associated with airway inflammation and can be experimentally induced by various inflammatory agents such as LPS [22], neutrophil elastase [27], cathepsin B [23], IL-4 [25], IL-9 [28], and IL-13 [29,30].

The exact mechanism of GCH, however, may differ in the experimental models. Neutrophils or eosinophils have been implicated in the induction of GCH in some animal models [30,31]. Neutrophils and eosinophils depleted rats using CPA or specific antibodies inhibit granulocyte in agarose plug-induced and IL-13-induced GCH model [29,31]. The epidermal growth factor receptor cascades are showed to be involved in underlying mechanism of the neutrophils – induced GCH [29,31]. However, in the present study we showed that depletion of these inflammatory cells by pretreatment with CPA similar dose used in the previously study [29,31] did not prevent TiO_2_-induced GCH (Figure 4). Because cyclophosphamide effectively suppressed the number of neutrophils and eosinophils in peripheral blood (data not shown) and airways in the present study although not complete (Figure 4), our data indicates that these inflammatory cells may be not responsible for the TiO_2_-induced GCH. The dissociation of GCH from airway eosinophilia has been well documented in murine asthma models, in which anti-IL-5 (TRFK-5) [32], or IL-5 deficiency [33] reduced airway eosinophilia without affecting the induction of GCH. Therefore, depending on the experimental models investigated, the induction of GCH may not require neutrophils and eosinophils. Furthermore, IL-13 is known to induce GCH without any help of other inflammatory cells [24] and has been clearly shown to play a single, common pathway by which GCH is induced by CD4+ cells and IL-9 [34]. This process needs IL-4 receptor alpha, but not IL-4 or IL-5 [33,34]. These data suggested a possibility that IL-13 is also involved in the particle – induced GCH.

In the present study, the levels of IL-13 in BAL fluids increased after TiO_2 _instillation concomitantly with the development of GCH and the increase of IL-13 was completely abolished by pretreatment with DEX (0.25 mg/Kg), but not by that with CPA (Figure 4). These results suggest that the elevation of IL-13 may be associated with particles such as TiO_2_-induced GCH without any assistance of neutrophils or eosinophils. The in vivo effect of dexamethasone has been also demonstrated in allergic asthma model [35]. Dexamethasone (4 mg / kg) effectively abolishes allergic airway inflammation in mice by suppression of IL-13 m-RNA and protein expression [35]. The exact biochemical mechanism of GCH induction by IL-13 is not fully understood. One possible explanation is that IL-13 converts the bronchial epithelium from an absorptive to a secretory phenotype through loss of an amiloride-sensitive current and an increase in calcium-sensitive apical anion conductance [36]. The increase in apical anion conductance in the airway epithelium is most likely due to the ability of IL-13 to induce expression of hCLCA1/mCLCA3, which encodes a calcium-activated chloride channel. This channel is necessary and sufficient for the development of GCH and mucus hypersecretion in some experiments [37].

Besides Th2 cells, IL-13 is produced by mast cells, eosinophils [38,39], and macrophages [40]. Since IL-13 was not decreased in rats of which eosinophils depleted by pretreatment of CPA (Figure 4), we can exclude eosinophils as the source of IL-13. Interestingly, serial thin section slides revealed that the IL-13 positive cells are mast cells, as shown by staining with toluidine blue. Also, we found the significant correlation between the IL-13 (+) rate of mast in tissue, concentration of IL-13 in BAL fluid and Muc5ac positive cells (Figure 5 and table 1). Based on these data, mast cells may be the cellular source for IL-13 present in the airways of TiO_2_-treated rats. It is well known that mast cells produce IL-13 when stimulated with antigen [39] and that the synthesis can be suppressed by dexamethsone [20]. Our finding showed that TiO_2 _instillation increased the numbers of IL-13 expressing mast cells and Muc5ac (+) goblet cells, both of which were decreased by dexamethsone pretreatment is a novel finding to our knowledge. It is not known whether TiO_2 _– induced IL-13 overproduction is specific to TiO_2 _or generally related to other particulates. However, base on the findings of particles such as diesel exhaust particles or carbon black particle – induced the deviation to Th2 environment in antigen sensitized lung [11,12], TiO_2 _– induced GCH via over production of IL-13 may be a general finding attributed to the particulate matters, but it remains unproven.

## Conclusion

We demonstrated that a single intratracheal instillation of TiO_2 _particles induces GCH and Muc5ac gene expression within 24 h in rats, and that this process may be associated with elevated amount of IL-13 derived from mast cells. The present study may provide experimental evidences to support that patients with chronic airway disease may aggravate their symptoms and airway functions in the heavily polluted environment of particulate matters.
